# Retinoic Acid Receptor γ Activity Plays a Critical Role in Regulating Early Mouse Gastruloid Development

**DOI:** 10.3390/ijms27093995

**Published:** 2026-04-29

**Authors:** Jide T. Olanipekun, Benjamin Edginton-White, Caitlin McQueen, Geoffrey Brown, William E. B. Johnson

**Affiliations:** 1 Chester Medical School, University of Chester, Chester CH1 4BJ, UK; 1722785@chester.ac.uk; 2 Department of Cancer and Genomic Sciences, School of Medical Sciences, College of Medicine and Health, University of Birmingham, Birmingham B15 2TT, UK; b.edginton-white@bham.ac.uk; 3 Biological Sciences, University of Chester, Chester CH1 4BJ, UK; c.mcqueen@chester.ac.uk; 4 Department of Biomedical Sciences, School of Infection, Inflammation, and Immunology, College of Medicine and Health, University of Birmingham, Birmingham B15 2TT, UK

**Keywords:** all-*trans* retinoic acid, retinoic acid receptor γ, embryonic development, gastruloid, axial patterning, cell fate, cell differentiation

## Abstract

Regulation of all-*trans* retinoic acid (ATRA) signaling is crucial to early embryonic development. Embryonic stem (ES) cell-derived gastruloids mimic normal development in response to the Wnt/β-catenin agonist CHIR9901, and this study has examined the importance of the activities of RAR (retinoic acid receptor) α and γ to gastruloid development. Expression of retinoic acid receptor (RAR)γ within developing gastruloids was spatially restricted to primitive cells that co-expressed ES cell and early progenitor cell markers, i.e., Nanog, Sox2, and Oct4. In contrast, RARα expression was ubiquitous. mRNAs for the key enzymes involved in ATRA synthesis (Aldh1a2) and degradation (Cyp26a1) were not seen in cells that expressed RARγ. Treatment of ES cell-derived gastruloids with physiologically relevant (10 nm) levels of ATRA or with a highly selective RARγ agonist blocked normal developmental processes, preventing symmetry-breaking and axial elongation. This was not seen following treatments with an RARα agonist, where there was a tendency for enhanced axial elongation. Brachyury (TBXT) immuno-positive cells localized in the posterior end of elongated gastruloids in control- and RARα agonist-treated cultures, with Sox2 immuno-positive cells seen more widely, whilst both TBXT and Sox2 immuno-positive cells were randomly distributed throughout ATRA- and RARγ agonist-treated gastruloids. Concurrent treatment of gastruloids with 10 nm ATRA and 100 nm of an RARγ antagonist partially abrogated the ATRA-mediated block to axial elongation. Conversely, 10 nm RARγ antagonist treatments were associated with the formation of multi-axis gastruloid elongations, with comparatively little effect seen after treatments with an RARα antagonist. These findings reveal that RARγ plays a crucial role in the development of embryonic tissues.

## 1. Introduction

A small and defined set of signaling pathways is used by the embryo during early development, which, when integrated over time, orchestrate axial patterning and morphogenesis to unfold the body plan. All-*trans* retinoic acid (ATRA), the major active metabolite of vitamin A, is a key signaling molecule. It contributes to many stages of development to regulate stem cell fate, axial development [1] and the generation of differentiated tissues, e.g., neuronal, cardiac, pancreatic, lung, and the eye [2,3]. ATRA activity is undetectable in the mouse embryo prior to embryonic day (E) 7.5 (approximately); however, following the formation of the mesoderm, ATRA activity occurs along the primitive streak, throughout the posterior portion of the embryo, and within the nascent mesoderm [4]. At later developmental stages, its activity is reduced in the tailbud, present in all tissues of the embryo trunk up to the boundary between the hindbrain and the first somite, and within optic lobes and the surrounding mesenchyme in the prospective head region ([4], reviewed in [5]).

A correct balance regarding the spatial and temporal distribution of ATRA synthesis and its activity is critical to the proper embryonic development [6,7,8,9], as environmental, genetic, or experimental perturbations that target ATRA signaling (either positively or negatively) cause wide-ranging abnormalities [6,10]. Accordingly, tissue access to ATRA within the developing embryo is tightly regulated through the activity of enzymes that control its synthesis from vitamin A, i.e., through retinol dehydrogenase (Rdh10) and aldehyde dehydrogenase (Aldh1a2) activity and its catabolism, through cytochrome P450 (Cyp26a1) activity [5]. Prior to gastrulation, Cyp26a1 is expressed throughout the embryo as a protective barrier against maternal ATRA [11]. By E7.25, Cyp26a1 expression occurs in the primitive streak following its progression anteriorly by E7.5 [12], and by E9.0 is confined to the tailbud [13]. Aldh1a2, on the other hand, the key enzyme in the final formation of ATRA, is first detected in the primitive streak and nascent mesoderm of E7.5 embryos, co-localizes with ATRA signaling, and forms a distinct signaling boundary with the expression of Cyp26a1 [14]. The ATRA concentration gradients that are established by the reciprocal actions of Rdh10, Aldh1a2 and Cyp26a1 are constantly modified throughout embryo development, and the changing gradients regulate signaling from other pathways that orchestrate morphogenesis also in a spaciotemporal manner, as provided, for example, by Wnts, transforming growth factors (TGFs) and fibroblast growth factors (fgfs) [15,16].

ATRA is the ligand for the three main isoforms of the retinoic acid receptors (RARs: RARα, RARβ and RARγ). They bind to the *cis*-acting response elements of ATRA target genes as a heterodimer with retinoid X receptor, and changes to transcription of downstream developmental genes occur following the engagement of ATRA with RARs [17]. The gene expression pathways that are ATRA-regulated via RARs are essential for normal embryonic development and cellular differentiation [10,18,19]. Embryonic stem (ES) cells, derived from the inner cell mass of the preimplantation blastocyst, have the capacity to differentiate into all cell and tissue types of the embryo. Therefore, they are an attractive model to study stem and progenitor cell-fate decisions and differentiation during early embryonic development [20]. When 200–300 ES cells are aggregated in 3D suspension cultures, they form spheroids, and when pulsed with the Wnt/β-catenin agonist CHIR99021 (CHIR) between 48 and 72 h, they recapitulate many aspects of early development [21,22]. After around 120 h of culture, the ES cells form so-called ‘gastruloids’, wherein 3D ES spheroids progressively break symmetry, polarize gene expression, can form three orthogonal axes (anteroposterior, dorsoventral, and mediolateral), and undergo axial elongation [21,22,23]. Pulsing with CHIR is essential for consistently reproducible axial elongation, but this is blocked by treating mouse and human gastruloid cultures with 0.4–33 nm concentrations of ATRA [24]. However, there is some confusion regarding the effect of ATRA *per se* on axial elongation. Other researchers generated human gastruloids using a combination of CHIR, the extracellular matrix components in Matrigel, and treated them with two different concentrations of ATRA exogenously at different times, i.e., with 500 nm ATRA in the earlier stages of ES spheroid formation (0–24 h) and 100 nm ATRA in the later stages of gastruloid elongation (48–120 h). This resulted in the robust formation of elongated gastruloids with neural tubes and somite-like structures [25]. Notably, 500 nm is a pharmacological level of ATRA.

The roles of physiological levels of ATRA (approximately 5–20 nm) [26,27,28] in determining ES cell fate during embryonic development are of particular interest, and moreover, the roles of individual RAR isoforms remain unclear from the experimental use of non-physiological levels of ATRA. The concentrations of ATRA used in many studies are generally not within the physiological range. For example, whilst pharmacological concentrations of ATRA (0.5–5 μm) have been used routinely to induce neuronal differentiation [25,29], 1.6 μm ATRA was shown to increase the presence of more primitive 2-cell-like totipotent cells (2CLCs) in ES cell cultures, which was largely due to activation of RARγ [30], and a lower level of ATRA (100 nm) induced the expression of mesodermal marker genes within mouse ES cells [31]. As gastruloids provide a highly tractable in vitro model for developmental studies, here we have examined the expression of RAR isoforms within the cell compartments in gastruloids to determine their potential association with developmental processes. Previously, we developed synthetic retinoids that were shown in transactivation studies to be highly specific for each of the RARs in the nm range [32]. Therefore, we have combined information provided by bioinformatic analyses that examined the presence of RARs in developing gastruloids with findings for the influence of 10 nm treatments of ATRA and of highly specific RAR agonists and antagonists on gastruloid development. This enabled us to examine how signals provided by the activities of RARα and RARγ, as governed by the spatial availability of ATRA, and Wnt/β-catenin signaling, are integrated to control tissue patterning and tissue formation. We show that RARγ activity plays a critical role in determining the progression of early embryonic development.

## 2. Results

### 2.1. The Expression of RARα and RARγ During Gastruloid Development

**Figure 1:**
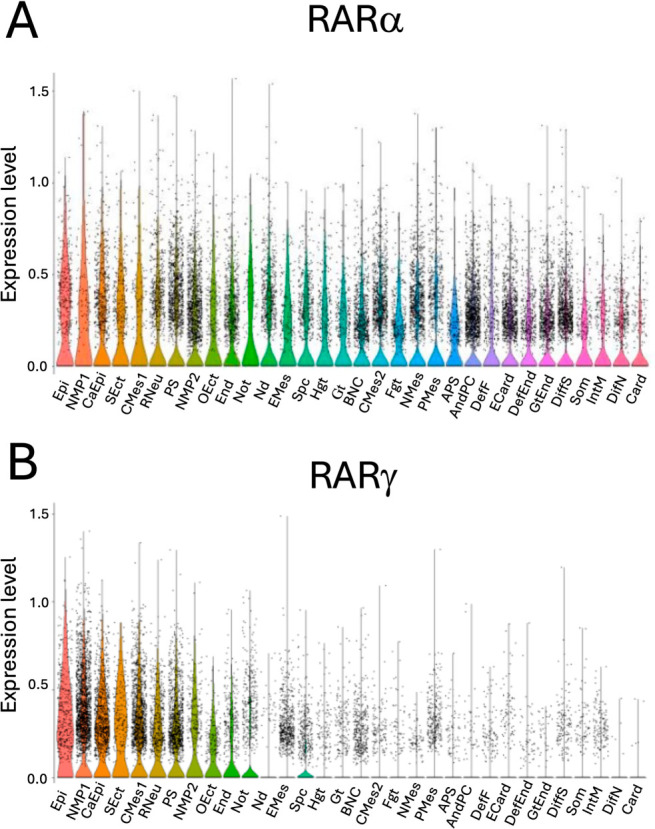
Expression of RARα and RARγ within mouse ES cells in gastruloid culture. Single-cell mRNA-seq datasets for gastruloids across four time points (days 4–7) were obtained from [33]. Using the previously identified cell type clusters [33], violin plots were produced to identify cell type-specific gene expression. (**A**) RARα mRNA was present in all the identified cell and tissue types. (**B**). RARγ mRNA was restricted to fewer cell and tissue types, and was strongly expressed in the NMPs, caudal epiblast, caudal mesoderm, rostral neuroectoderm, and primitive streak. Abbreviations: Epi: epiblast; NMP1: neuromesodermal progenitor; CaEpi: caudal epiblast; SEct: surface ectoderm; CMes1: caudal mesoderm; RNeu: rostral neuroectoderm; PS: primitive streak; NMP2: neuro mesodermal progenitors (mesoderm); OEct: oral ectoderm; End: endothelium; Not: notochord; Nd: node; EMes: early mesoderm; Spc: spinal cord; Hgt: hindgut; Gt: gut; BNC: brain and neural crest; CMes2: cardiac mesoderm; Fgt: foregut; NMes: nascent mesoderm; PMes: pharyngeal mesoderm; APS: anterior primitive streak; AndPC: hematoendothelial progenitors; DefF: definitive front; ECard: early cardiomyocytes; DefEnd: definitive endoderm; GtEnd: gut/visceral endoderm; DiffS: differentiated somite; Som: somite/sclerotome; IntM: intermediate mesoderm; DifN: differentiated neurons; and Card: cardiomyocytes.

Levels of mRNA for RARα and RARγ, as identified using single-cell RNA sequencing in days 4–7 of gastruloid development [33] (downloaded from the Gene Expression Omnibus, accession number: GSE158999), were correlated with the expression of genes that are associated with different cell and developing tissue types. These data are shown as violin plots in Figure 1. As shown, RARα expression was largely present across all cell and tissue types (Figure 1A). In contrast, RARγ expression was restricted across developing tissues and was highly expressed only in those primitive tissues that contained prominent stem/progenitor cell compartments, i.e., the epiblast, NMPs, caudal epiblasts, caudal mesoderm, rostral neuroectoderm, and the primitive streak (Figure 1B).

**Figure 2:**
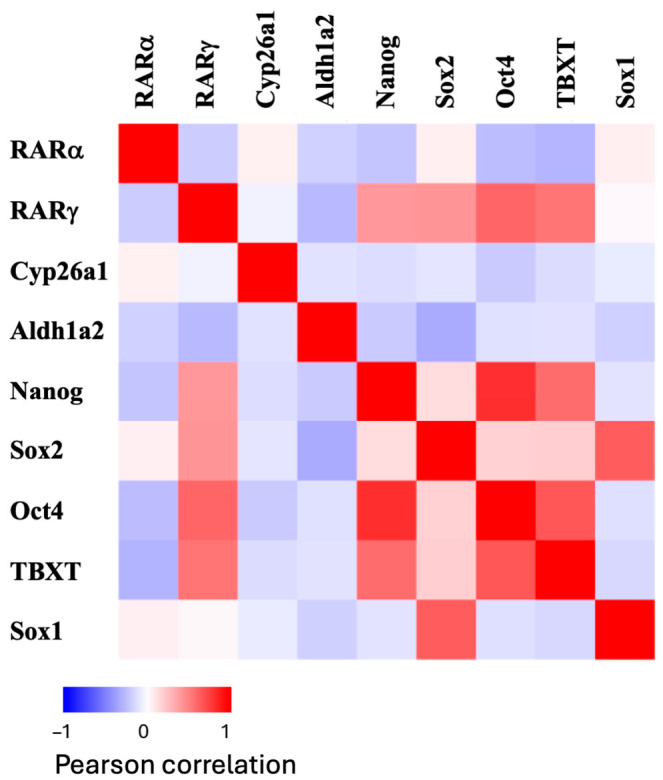
RARγ expression correlates with gene expression of stem cell/developmental transcription factors. The heatmap shows the Pearson rank correlation of gene pairs across all cell types and combined days based on the expression of genes in metacells. There is a positive correlation between RARγ mRNA levels with that of Nanog, Sox2, Oct4, and TBXT mRNAs. In contrast, there was no marked correlation of RARα mRNA expression with these genes in all cells. For the enzymes involved in ATRA synthesis (Aldh1a2) and degradation (Cyp26a1), the cells that expressed RARγ mRNA did not express mRNAs for these enzymes. TBXT (T-box transcription factor, commonly known as brachyury) is essential for gastruloid elongation. Nanog, Sox2 (sex determining Y-box2), and Oct 4 (octomer-binding transcription factor 4) are transcription factors that are important to self-renewal/the pluripotency of stem cells. Sox1 (SRY-box transcription factor) is required to maintain neural stem cells.

Next, we explored the phenotype of cells that expressed RARα and RARγ both in relation to each other and the expression of the ATRA metabolizing enzymes, Cyp26a1 and Aldh1a2, and also in relation to pluripotent stem cell and development regulatory-associated transcription factors, i.e., Nanog, Oct4, Sox2, Sox1, and brachyury (TBXT). A heatmap showing the Pearson correlation for co-expression of these various gene pairs across all cells in gastruloids during days 4–7 is shown in Figure 2. RARα and RARγ were not expressed in the same cells. There were no marked correlations between RARα expression and the expression levels for the pluripotent stem cell genes, i.e., Nanog or Oct4, nor with TBXT. In contrast, there were strong positive correlations between RARγ expression and Nanog, Oct4, as well as Sox2 and TBXT. RARγ expression did not correlate with Cyp26a1, Aldh1a2, and Sox1 expression. This analysis shows that the cells in gastruloids that express RARγ are primitive in nature and that they are dependent on exogenous ATRA levels for ATRA-regulated processes.

### 2.2. The Effects of ATRA and RARα and RARγ Specific Agonists and Antagonists on Gastruloid Development

Having observed that RARs were differentially expressed in developing gastruloids, and that the cells that expressed RARγ do not express the enzymes required for ATRA synthesis or degradation, we examined the effects of treating gastruloids with physiological levels of exogenous ATRA and RAR isoform-specific synthetic retinoids. When aggregates of ES cells were stimulated with the Wnt/β catenin agonist CHIR from 48 to 72 h of culture in N2B27 media, which is the standard culture condition for the generation of gastruloids, they broke symmetry and underwent axial elongation by 120 h (n = 51 gastruloids). This was also seen for DMSO-treated cultures, i.e., carrier controls in N2B27 media (n = 62 gastruloids). However, when 10 nm ATRA was also added to CHIR-stimulated ES aggregates at 48–72 h, there was a complete block in symmetry breaking and no axial elongation at 120 h (n = 61 gastruloids). Treatments with 10 nm of the RARγ agonist AGN205327 over the same period had the same effect, i.e., there was a complete block in symmetry breaking and no axial elongation (n = 29 gastruloids). All the ATRA- and RARγ agonist-treated cultures were spheroidal at 120 h. Conversely, treatments of CHIR-stimulated ES aggregates with 10 nm of the RARα agonist AGN195183 from 48 to 72 h led to symmetry breakage, and the gastruloids had undergone axial elongation by 120 h (n = 51 gastruloids). In addition, axial elongations were seen most prevalently following gastruloids treatments with 10 nm of the RARα antagonist AGN196996 (n = 46 gastruloids) or 10 nm of the RARγ antagonist AGN205728 (n = 56 gastruloids). There were significant increases in the frequencies of spheroid morphologies and significant decreases in the frequencies of elongated morphologies in the ATRA- and RARγ agonist-treated groups versus the DMSO control group (*p* < 0.001, Chi-Squared tests). These were the only significant differences seen in any of the morphological criteria from the DMSO control group.

**Figure 3:**
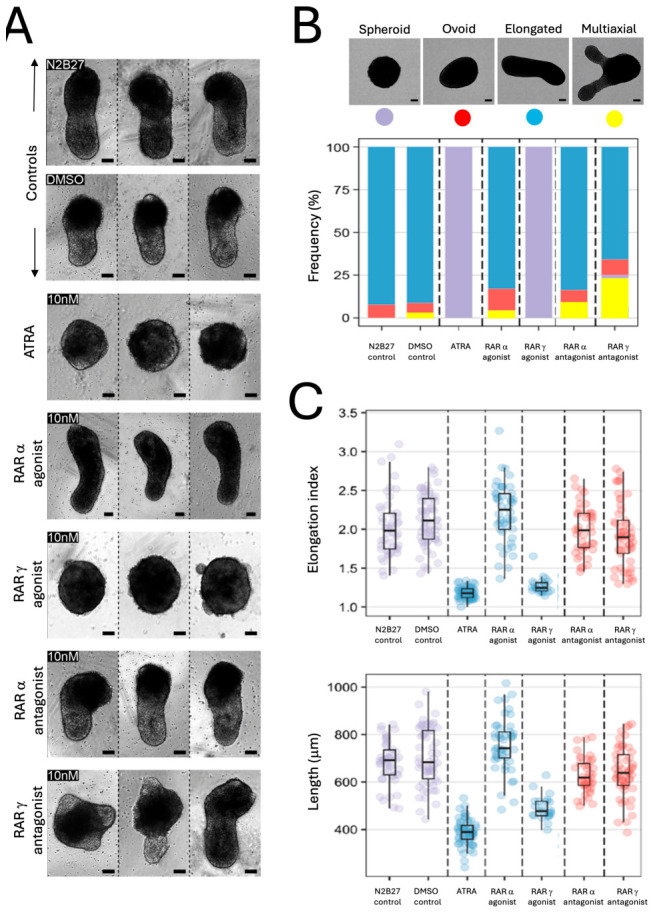
Physiological levels of 10 nm ATRA and 10 nm RARγ agonism blocked gastruloid axial elongation. (**A**). Representative brightfield images from at least three independent samples (biological repeats) are shown for gastruloids at 120 h that had been cultured in N2B27 medium for 48 h and stimulated with a pulse of 3 μm CHIR between 48 and 72 h, along with gastruloids treated identically but with either DMSO or 10 nm of ATRA or 10 nm of specific agonists and antagonists of RARγ or RARα. The medium was replaced with fresh N2B27 without any supplementation at 72 and 96 h. Gastruloids were imaged and fixed at 120 h. ATRA and selective RARγ agonism completely blocked axial elongation, whereas RARα agonism did not. All scale bars are 100 μm. (**B**). The proportions of gastruloids that were classified as elongated (blue), ovoid (red), spheroid (purple) or multiaxial (yellow) in each experimental group at 120 h of culture. (**C**). The elongation indices and lengths of gastruloids in each experimental group at 120 h of culture. There were significant differences in these measures between the DMSO carrier control group and the 10 nm ATRA- and 10 nm RARγ agonist-treated groups (*p* < 0.001), and between the 10 nm RARα agonist-treated group (*p* < 0.05) versus the N2B27 control group, but not versus the DMSO carrier control group (Kruskal–Wallis with Mann–Whitney *U* tests). Data are shown as box and whisker plots, with values pooled from n = 3 independent experiments. The number of gastruloids examined in each group is given in the main text. The arrows delineate the control N2B27 and DMSO groups.

ES aggregates treated with either 10 nm ATRA or 10 nm RARγ agonist had marked and significant reductions in their measured elongation indices (indicating gastruloid shape) and overall lengths at 120 h compared with the gastruloids in standard inductions in N2B27 medium and in the DMSO carrier control cultures (both *p* < 0.001 versus DMSO controls, Kruskal–Wallis with Mann–Whitney *U* tests). There was a moderate increase in the elongation index and length of 10 nm RARα agonist-treated gastruloids at the same time point, which was significantly greater than the N2B27 control cultures (*p* < 0.05, Kruskal–Wallis with Mann–Whitney *U* test), but not the DMSO control cultures. Most of the CHIR-stimulated ES aggregates treated with 10 nm of the RARα antagonist or of the RARγ antagonist from 48 to 72 h broke symmetry and underwent axial elongation by 120 h. It was also noteworthy that there was a tendency for some gastruloids in all conditions that included DMSO, i.e., all except the N2B27 control cultures, to form multiaxial elongations, and that this phenotype was most prevalent in the 10 nm RARγ antagonist-treated cultures, where 23% of the RARγ antagonist-treated gastruloids formed multiaxial elongations, compared with only 5% of the DMSO control group, which was not a significant difference (Figure 3).

**Figure 4:**
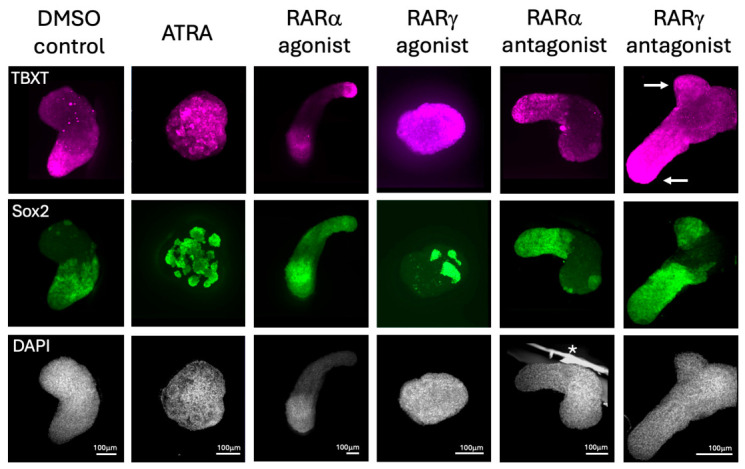
TBXT and Sox2 immunopositivity in gastruloids treated with ATRA- and RAR-specific agonists and antagonists. Representative fluorescence images are shown after TBXT and Sox2 immunohistochemistry and confocal microscopy using projected images of z stacks of gastruloids at 120 h that had been stimulated with 3 μm CHIR between 48 and 72 h, along with either DMSO (carrier control cultures) or with 10 nm ATRA or 10 nm of agonists and antagonists specific for RARγ or RARα. As shown, TBTX localized in the posterior tips of gastruloids that had successfully elongated, with Sox2 immuno-positive cells present in adjacent tissue. In contrast, clusters of TBXT and Sox2 immuno-positive cells were seen randomly distributed in the ATRA- and RARγ agonist-treated ES cultures, which remained as spheroids. Interestingly, TBXT and adjacent Sox2 immunopositivity were seen in the tips of outgrowing branches of those gastruloids that underwent multiaxial elongation (arrowed in RARγ antagonist-treated culture). There were n = 3 independent experiments (biological repeats) with totals of 8, 7, 10, 9, 8, and 7 gastruloids stained for each marker in the DMSO control, ATRA-treated, RARα agonist-treated, RARγ agonist-treated, RARα antagonist-treated, and RARγ antagonist-treated groups, respectively. All scale bars are 100 μm (* artifact).

We next examined the presence of key transcription factors that are known to localize differentially during gastruloid formation. Immunostaining showed that TBXT was present at 120 h, localized to the posterior end of gastruloids in DMSO control cultures and RARα agonist-, RARα antagonist-, and RARγ antagonist-treated cultures, all of which had elongated to a similar extent. Conversely, TBXT immunopositivity was seen to be localized to clusters of cells throughout the ATRA- and RARγ agonist-treated gastruloid cultures, which were spheroidal at 120 h after treatment. Hence, it was clear that TBXT immune-positive cells were only localized in those gastruloids that had broken symmetry and undergone axial elongation. Interestingly, we also observed that TBXT was seen in the tips of multiaxial elongations, in many but not all cases. Immunopositivity for Sox2 was seen in all gastruloids, often in the proximity of TBXT cells, but without direct co-localization. In the ATRA- and RARγ agonist-treated cultures, Sox2 was seen in groups of clustered cells, suggesting compartmentalization, but the distributions of these clusters appeared random throughout the spheroids. There were no clear differences in the intensity of TBXT or Sox2 in any of the experimental groups, suggesting a similar level of expression of these transcription factors (Figure 4).

### 2.3. The Inhibitory Effects of ATRA on Gastruloid Axial Elongation Were Partially Abrogated by RARγ Specific Antagonism

**Figure 5:**
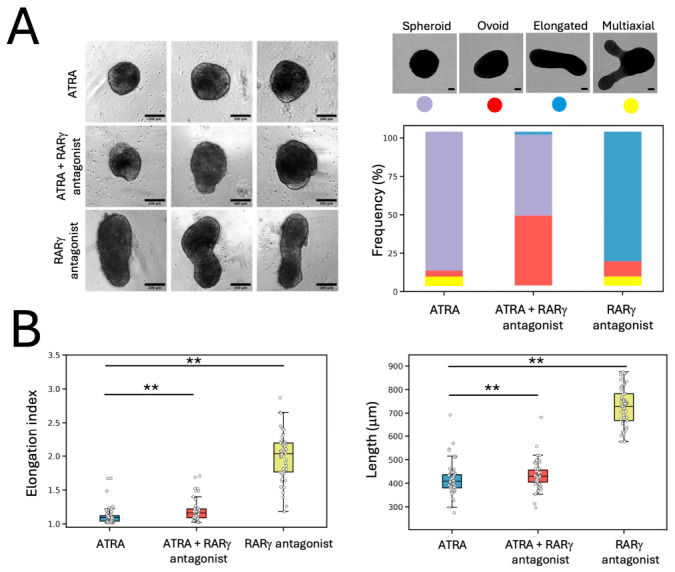
ATRA-mediated block in gastruloid axial elongation was partially abrogated by RARγ antagonism. (**A**). Left panel: Representative brightfield images are shown of gastruloids at 120 h that had been stimulated with 3 μm CHIR between 48 and 72 h and treated at the same time with 10 nm ATRA or with 10 nm ATRA plus 100 nm of an RARγ antagonist or with 100 nm RARγ antagonist. All scale bars are 200 μm. Right panel: the frequency of gastruloid cultures under these conditions that were spheroid (purple bars), ovoid (red bars), elongated (blue bars), or multiaxial (yellow bars). As shown, and as detailed in the main text, there was a marked increase in the presence of ovoid gastruloids when 100 nm RARγ antagonism was combined with 10 nm ATRA. (**B**). The elongation indices and lengths of gastruloids in each experimental group at 120 h of culture. There were significant differences in these measures between the 10 nm ATRA-alone versus the 10 nm ATRA plus 100 nm RARγ antagonist-treated group, between the 10 nm ATRA-alone versus the 100 nm RARγ antagonist-treated group, and between the 10 nm ATRA plus 100 nm RARγ antagonist-treated group versus the 100 nm RARγ antagonist-treated group (** *p* < 0.01) (Kruskal–Wallis with Mann–Whitney *U* tests). Data are shown as box and whisker plots, with values pooled from n = 3 independent experiments.

Finally, in separate experiments, we tested whether the effects of 10 nm ATRA-treatment could be abrogated by concurrent treatments with the RARγ antagonist, using a 10-fold concentration of 100 nm of the antagonist. We used this level of the RARγ antagonist to ensure reversal of the ATRA agonistic action. Markedly more ovoid morphologies were seen at 120 h regarding the gastruloids treated with ATRA plus RARγ antagonist, as compared with the ATRA alone-treated group, in more than 50% of all gastruloids. In these experiments, 47 of 52 of the 10 nm ATRA-treated gastruloids were spheroid, three were ovoid, none were elongated, and two were multiaxial. This compares with gastruloids treated with 10 nm ATRA plus 100 nm RARγ antagonist, where 29 of 55 gastruloids were spheroid, 25 were ovoid, one was elongated and none were multiaxial. In the 100 nm RARγ antagonist alone-treated group (n = 51), none of the gastruloids were spheroid, five were ovoid, 43 were elongated and three were multiaxial. There were significant differences (*p* < 0.001, Chi-Squared tests) in the frequencies of spheroid and ovoid gastruloids only in the 10 nm ATRA-treated versus 10 nm ATRA plus 100 nm RARγ antagonist-treated groups. Furthermore, there was a subtle but nonetheless significant difference (*p* < 0.01, Kruskal–Wallis with Mann–Whitney *U* tests) in both the elongation index and the overall length of the gastruloids in the 10 nm ATRA plus 100 nm RARγ antagonist-treated group compared with the 10 nm ATRA alone-treated group. Treatments with 100 nm RARγ antagonist alone were clearly associated with axial elongation, as seen in control groups (Figure 5).

## 3. Discussion

We have explored the roles of specific RARs in the regulation of retinoic acid signaling in early embryonic development using ES-derived gastruloids as a model system and highly specific RAR agonists and antagonists at physiological concentrations. We show that RARγ is expressed specifically in stem/progenitor cell compartments of developing gastruloids, whereas the expression of RARα is widespread. We also show that the enzymes needed for ATRA synthesis, Aldh1a2, and catabolism, Cyp26a1, are not expressed by RARγ-positive cells. Further, we have shown that treatments with physiological levels of exogenous ATRA or with an RARγ-specific agonist blocked the development of mouse ES-derived gastruloids in response to stimulation of the Wnt/β catenin signaling pathway. Axial elongation was ablated in these conditions. Conversely, 10 nm RARα agonist treatments of gastruloids tended to increase axial elongation. These data show that the effects of ATRA in blocking axial elongation during gastruloid development are mediated through RARγ activity. In contrast to the action of the RARγ agonist, the gastruloids that were treated with 10 nm of the RARγ antagonist had differentiated, having mostly broken symmetry and elongated. However, some had multiple axes, perhaps indicating a role for RARγ in axis specification. Whether this is the case requires further investigation.

Previously, we showed that treating zebrafish embryos at 4 h post-fertilization with 10 nm of the RARγ-specific agonist AGN205327, in the absence of exogenous ATRA, disrupted the formation of many tissues, which was not seen following treatments with the RARα-specific agonist AGN195183 [18]. A block to stem/progenitor cell differentiation led to severe truncation of the embryonic zebrafish, with fewer somites in the tail, loss of the most posterior regions of the trunk, including the caudal fin, and a lack of pectoral fin, reduced craniofacial bones and anterior neural ganglia. The RARγ agonist also blocked caudal fin regeneration when the fin was transected at 2–3 days post-embryo fertilization. RARγ agonism did not lead to a loss of the Tbx-5^+^ lateral plate mesodermal stem/progenitor cells, because these cells were still present after RARγ agonist treatment, and the block to pectoral fin development was reversible if the RARγ agonist in the zebrafish water was washed out or if an RARγ antagonist was added to the water. Furthermore, washouts of the RARγ agonist or co-treatments with an RARγ antagonist restored caudal fin regeneration. Here, we found that TBXT and Sox2 immuno-positive cells remained in ATRA- and RARγ agonist-treated ES-derived gastruloids, which, similarly to zebrafish, had been blocked in development. TBXT and Sox2 are key regulators of gastruloid development [34], with TBXT activity in particular determining symmetry breaking and the extent of axial elongation [21,35]. The presence of TBXT and Sox2 immuno-positive cells in the ATRA and RARγ agonist-treated cultures suggested that these gastruloids maintained a capacity for development. Furthermore, we found that adding an excess level of RARγ antagonist at the same time as ATRA resulted in a partial abrogation of the ATRA-mediated block in axial elongation. Therefore, our current findings, along with those previously reported for zebrafish [18], support the view that the activation of RARγ by physiological levels of ATRA functions to block stem/progenitor cell differentiation in developing tissues.

In addition to promoting the formation of totipotent 2CLCs in mouse ES cultures [30], RARγ ligation was shown repeatedly to have a positive effect on the establishment and/or maintenance of naïve-state pluripotency during the generation of induced pluripotent cells (iPSCs). Transgene expression of the orphan nuclear receptor, liver receptor homolog 1, reprogrammed Oct4-GFP epiblast stem cells into iPSCs and, post recovery from transgene expression, the addition of 0.1 nm ATRA, to activate RARγ, into ATRA-free medium enhanced cellular reprogramming, whereas RARγ antagonism had a deleterious effect; further, this reprogramming enhancement required the presence of ATRA and was dependent on the presence of liganded RARγ [36]. For mouse embryonic fibroblasts (MEFs), adding transgene expression of RARγ and liver receptor homolog 1 to enhanced expression of Oct4, Sox2, c-Myc, and Klf4 promoted MEF reprogramming to iPSCs [37]. RARγ ligation had a positive effect on the generation of iPSCs derived from human dermal fibroblasts by transgene expression of Oct4, Sox2, Klf4, L-Myc, and p53, through the additional use of the RARγ agonist CD437 and the liver receptor homolog 1 agonist RJW101, along with inhibitors of glycogen synthase kinase-3 and mitogen-activated protein kinase kinase 1–2 [38]. These findings suggest that RARγ activation promotes the generation and/or proliferation of pluripotent stem cells, as well as their maintenance in an undifferentiated state.

It is possible, however, that the regulatory role of RARγ activity extends to stem/progenitor cell populations more widely than in embryonic development and pluripotency. In studies by Shimono and colleagues [39], the RARγ agonist NRX204647, and ATRA at concentrations of 3 nm to 30 nm, blocked ectopic bone formation in a transgenic model of fibrodysplasia ossificans progressiva. Bone morphogenetic proteins (BMPs) drive mesenchymal stem cell (MSC) differentiation towards chondrogenesis and osteogenesis in the developing limb [40] as well as in fracture repair [41], and in vitro treatment of mouse MSCs with the RARγ agonist rendered these cells unresponsive to BMP-2. The levels of Smad proteins were decreased within the RARγ agonist-treated MSCs [39]. Moreover, liganded RARγ interferes directly with Smad signaling as reported from transfection studies of lung fibroblast and HepG2 liver cells that made use of a Smad3 reporter, overexpression of individual RARs, and selective agonists and antagonists [42,43]. From these studies, RARγ bound to Smad3, whereas liganded RARα and RARγ both applied a break to cell responsiveness to transforming growth factor (TGF)β, and non-liganded RARα and RARγ enhanced their responsiveness; inactive RARγ was more potent than RARα in modulating such TGFβ-dependent Smad3-mediated signaling. From the above, it is known that RARγ plays a non-canonical role as a cofactor to Smads in the regulation of TGFβ signaling.

Regarding the generation of iPSCs from epiblast stem cells, liganded RARγ had exerted a negative effect on Wnt signaling [36], and the enhancement of reprogramming of human dermal fibroblasts by liganded RARγ was attributed to a positive influence on TGFβ signaling [38]. Appropriate Wnt/β catenin signaling is essential in embryo axis formation and tissue organogenesis [43], and multiple Wnts and their receptors are regulated by RARs [44,45]. TGFβ signaling family members direct early stem/progenitor cell-fate decisions as master regulators of organogenesis [46]. For some cancer cells, RARγ is predominantly cytoplasm and activation of Wnt/β-catenin signaling has been reported for cholangiocarcinoma [47] and colorectal cancer cells [48]. Hence, in our studies, agonizing RARγ may have interfered with Wnt/β catenin and/or TGFβ signaling to block gastruloid development.

However, any RARγ-mediated events that block gastruloid development may be more complex than direct effects on Wnt/β-catenin or TGFβ signaling. The genes that are regulated by RARγ within gastruloids are not known. Hundreds of genes were seen to be regulated, either directly or indirectly, from studies of ATRA-induced differentiation of F9 mouse ES cells [49]. From studies of RARγ knockout in human squamous cell carcinoma cells, the target genes identified play key roles in squamous cell differentiation [50]. For knockout cells without added RAR ligand, transcripts for NOTCH1, NOTCH3, the NOTCH ligands JAG2 and DLL1, and a broad group of genes pertaining to cell identity and extracellular matrix communication were reduced. *RARG*, *PPARG*, and *RXRA* were expressed at a higher level in the knockout cells and, therefore, repressed by RARγ. Control of *RXRA* expression is important because RXRα has multiple dimerization partners. Even so, it is important to bear in mind that RARγ acts within cells in a manner that is context-dependent.

How agonism of RARγ activity blocks gastruloid development is still unclear because there is uncertainty regarding whether the outcome from treating gastruloids with the RARγ agonist relates to an action against ES cells and/or committed progenitor cells. Uncertainty in this regard confounds making clear connections between the many primitive/progenitor populations that clearly express RARγ and the phenotypes that were observed for the gastruloids that were treated with the RARγ agonist. Moreover, we do not yet know about the presence/prevalence of the primitive/progenitor populations within the RARγ-agonist-treated gastruloids. Regarding a mode of action for the RARγ, many different regulatory roles have been identified. The multifaceted roles of RARγ include the direct regulation of gene expression and roles as a cofactor to other transcription factors and within the cytoplasm to modulate intracellular signaling. For the many cell regulatory events that involve RARγ, there is considerable crosstalk between pathways, for example, between Wnts/β-catenin and BMPs, TGFβ/Smad 3, and fgf signaling, and RARγ and other steroid hormone nuclear receptors. Therefore, a scheme to depict how RARγ agonism blocks gastruloid development is yet uncertain. Nonetheless, the varied roles of RARγ and crosstalk may ensure tight regulation of the behavior of stem/progenitors, whereby RARγ integrates multiple processes and findings to date highlight RARγ modulation of the responsiveness of stem/progenitor cells to extracellular signals that play a key role in governing changes to their behavior. This study has further highlighted that the activation level of RARγ plays a key role in determining whether stem cells progress to enable tissue formation and development.

A limitation of this study is that we have looked at a single window of gastrulation to examine the effect of the RARγ and RARα agonists at the time the gastruloids were pulsed with CHIR. Regarding future studies, examination of shorter windows regarding the exposure of gastruloids to retinoids and later windows, making use of removal or the RARγ antagonist to reverse the agonist action, should reveal dynamic regulatory information. Additionally, we do not yet know the precise influence of the RARγ antagonist on tissue development, which will require a detailed analysis of the extent to which various populations of cells are affected. We also have seen some moderate effect of RARα agonism in enhancing the elongation of gastruloids, which, although insignificant, should be explored further, as it is well known that ligated RARα plays a key role in regulating differentiation [19]. A further limitation may be that we performed these experiments using a mouse gastruloid model, whereby these experiments could have been performed in mice in vivo or using ex vivo models. Although it also may be considered useful to examine the effects of RARs in a mouse gastruloid system with a view to further research of human gastruloid systems, where in vivo or ex vivo models are not possible.

## 4. Materials and Methods

### 4.1. Single-Cell RNA-Seq Analysis

Single-cell RNA-seq data for gastruloids across 4 time points produced by REF were downloaded from the Gene Expression Omnibus (GEO, NCBI), accession number: GSE158999. The downloaded data was already aligned, clustered, and the cluster cell types identified as detailed in Rossi et al. [33]. The data was loaded into R v4.2.2 and analyzed using the Seurat package v4 [51]. Volcano plots showing gene expression for targets in different clusters were produced using the vlnplot function in Seurat.

For pairwise comparison of gene expression visualized as a Pearson correlation heatmap, the data were converted to metacell data. This helps to overcome the sparsity of the single-cell data and enables correlations to be calculated. Metacells were constructed using the hdWCGNA (version 20240201-foss-2022b-R-4.3.1) [52], and expression was normalized using the NormalizeMetacells function. Scatter plots for pairwise gene comparison with calculated Pearson correlation values were then plotted using the ggplot2 package.

### 4.2. Retinoids

The binding affinities and specificities of the selective and stable RARα and RARγ agonists and antagonists have been described previously [32]. Both agonists do not transactivate retinoid X receptors. RARβ shows some response to the RARγ agonist, but this only occurs at high, non-physiological doses, i.e., greater than 100 nm, and was not a confounding issue regarding the doses used in experiments. The retinoids were synthesized at the Shanghai Institute of Materia Medica. They were dissolved in dimethylsulphoxide (DMSO) at a concentration of 10 mM (stored at −20 °C), and this stock was diluted using culture medium to the required concentration. ATRA, which was also dissolved in DMSO, was purchased from Sigma-Aldrich (R2625, St. Louis, MO, USA).

### 4.3. Routine ES Cell Culture

Wild-type E14Tg2A mouse ES cells were seeded at a density of 1.2 × 10^4^ cells/cm^2^ on 0.1% (*v*/*v*) gelatin-coated flasks in GMEM (Gibco, Grand Island, NY, USA) supplemented with 15% fetal bovine serum (FBS; Gibco Cat. No. 10270-106), non-essential amino acids (Gibco; 11140035), sodium pyruvate (Gibco; 11360039), Glutamax™ (Gibco; 35050038), 2-mercaptoethanol (Gibco; 31350010), and LIF (QKine, Cambridge, UK; QK018); hereon referred to as ESL medium. Cells were cultured in a humidified incubator maintained at 37 °C with 5% CO_2_, and passaged every other day, with full medium changes on non-passage days. Cells were tested monthly and certified negative for mycoplasma.

### 4.4. Generation of Gastruloids and Treatments with ATRA and Synthetic Retinoids

Gastruloids were generated as previously described [21]. Briefly, 350 viable ES cells/well were plated in low-adherence U bottomed 96-well plates using a multichannel pipette in 40 μL N2B27 medium and left to aggregate for 48 h at 37 °C with 5% CO_2_, after which 150 μL of N2B27 medium supplemented with 3 μm CHIR was added with 10 nm ATRA or with specific 10 nm RAR agonists/antagonists or with/without the DMSO carrier control, where the DMSO was diluted to the highest concentration of ATRA or added synthetic retinoid of combinations. At 72 h and 96 h after aggregation, 150 μL medium was removed from each well using a multichannel pipette and replaced with fresh N2B27 medium minus the additional ATRA or retinoids. Samples were imaged by brightfield microscopy (see below) and fixed at 120 h.

### 4.5. Immunostaining

Cells grown as gastruloids were fixed in 4% (*v*/*v*) formaldehyde in phosphate-buffered saline (PBS) for half an hour at room temperature, after which they were briefly rinsed with PBS supplemented with 10% FBS and 0.2% Tween20 (PBSFT), followed by multiple hour-long incubations in PBSFT. Samples were then incubated with primary antibodies: goat anti-TBXT (PA5-46984; ThermoFisher, Waltham, MA, USA; 1:50) and rabbit anti-Sox2 (AB5603; Abcam, Cambridge, UK; 1:200) (diluted in PBSFT) and incubated overnight at 4 °C. Primary antibodies were then removed, samples rinsed briefly three times in PBSFT, followed by multiple hour-long washes in PBSFT. Samples were incubated with secondary antibodies: donkey anti-goat Alexa Fluor 568 (A-11057; Invitrogen, Carlsbad, CA, USA; 1:500) and donkey anti-rabbit Alexa Fluor 488 (A-21206; Invitrogen; 1:500) (diluted in PBSFT) and incubated overnight at 4 °C. DAPI was also included (1:1000) as a counterstain to mark nuclei. Following multiple rinses in PBS supplemented with 0.2% FBS and Tween 20, gastruloids were mounted between glass slides (No. 1 thickness) in Rapiclear, and adhesive spacers were used to ensure they were not compressed. Samples were stored at 4 °C until required.

### 4.6. Microscopy and Image Analysis

Live gastruloid brightfield images were acquired using a Nikon Ti-E inverted widefield microscope (Nikon, Tokyo, Japan) in a humidified incubator (37 °C, 5% CO_2_). Imaging was performed using a 20× long working distance phase-contrast objective (NA 0.35, Ph1) with a correction collar set to image through plastic. Transmitted light images were captured using a digital camera with an exposure time of 43 ms and recorded using Nikon-Elements software (NIS-Elements AR software, version 4.51.0; Nikon, Tokyo, Japan). For immunofluorescence analysis, fixed, stained and mounted gastruloids were imaged using an Andor Dragonfly spinning disk confocal system mounted on a Leica DMi8 inverted microscope (Leica Microsystems, Wetzlar, Germany), using a 25× water-immersion objective (0.95 NA). Fluorophores were excited sequentially using 405 nm, 488 nm, and 561 nm laser diodes, respectively, and emitted light reflected through 450/50 for DAPI, 525/20 for Alexa Fluor 488, and 620/60 for Alexa Fluor 568 (all Thermo Fisher, Loughborogh, UK) bandpass filters, respectively. Emitted light was captured using an iXon Ultra 888 EM-CCD camera (Andor Technology, Belfast, UK), with 1 × 1 binning, and recorded using Fusion software (Fusion Release 2.3). Z-stacks were captured at Nyquist sampling intervals. Microscopy images were processed in the ImageJ package Fiji (Release 2.17.0). The length and elongation index (length/largest circle that fits within the gastruloid area) were measured as previously described [21,53].

### 4.7. Statistical Analysis

At least 3 independent experiments (biological repeats) were performed, each with multiple technical repeats (minimum 3 repeats) for all analyses. Data were pooled, and normality testing was assessed using the Shapiro–Wilk test, and significance was determined using a non-parametric Kruskal–Wallis rank sum test and post hoc Mann–Whitney *U* tests using the Benjamini–Hochberg Procedure to correct for multiple comparisons. Significant differences between morphological criteria were determined using Chi-Squared tests, corrected with Bonferroni. All data analysis, statistical tests, and graphing were performed in RStudio (2023.12.1+402). Data have been shown as histograms and box and whisker plots with overlaid data points. *p*-values < 0.05 were considered significant.

## Data Availability

The original contributions presented in this study are included in the article. Further inquiries can be directed to the corresponding authors.
